# Three-Way Interactions in an Artificial Community of Bacterial Strains Directly Isolated From the Environment and Their Effect on the System Population Dynamics

**DOI:** 10.3389/fmicb.2019.02555

**Published:** 2019-11-13

**Authors:** Óscar Adrián Gallardo-Navarro, Moisés Santillán

**Affiliations:** Unidad Monterrey, Centro de Investigación y de Estudios Avanzados del IPN, Apodaca, Mexico

**Keywords:** microbial ecology, population dynamics, mixed bacterial colonies, mathematical model, antagonism

## Abstract

This work is motivated by previous studies that have analyzed the population ecology of a collection of culturable thermoresistant bacteria, isolated from the Churince lagoon in Cuatro Cienegas, Mexico. In particular, it is aimed at testing a hypothesis from a modeling study, which states that antagonistic and sensitive bacteria co-exist thanks to resistant bacteria that protect sensitive ones by forming physical barriers. We selected three different bacterial strains from the referred collection: one antagonistic, one sensitive, and one resistant, and studied the population dynamics of mixed colonies. Our results show that, although the proposed protective mechanism does not work in this case, the resistant strain confers some kind of protection to sensitive bacteria. Further modeling and experimental results suggest that the presence of resistant bacteria indirectly improves the probability that patches of sensitive bacteria grow in a mixed colony. More precisely, our results suggest that by making antagonistic bacteria produce and secrete an antagonistic substance (with the concomitant metabolic cost and growth rate reduction), resistant bacteria increase the likelihood that sensitive bacteria locally outcompete antagonistic ones.

## 1. Introduction

Bacteria have existed on Earth for more than 3.5 billion years (Schopf, 1993), and in such time they have colonized nearly every surface on the planet. In general, bacteria and other microorganisms form communities in which different species co-exist, share resources, and compete. Some important phenomena at planetary scale, like atmosphere oxygenation (Ohno, 1997) and biogeochemical cycles (Falkowski et al., 2008), are thought to be due to the action of bacterial communities. Bacterial communities also interact symbiotically with higher organisms (Fisher and Long, 1992; Currie, 2001; Anand and Sripathi, 2004; Turnbaugh et al., 2007), and destabilization of these communities causes diseases (Turnbaugh et al., 2007; Petrof et al., 2013; Ofosu, 2016).

Despite the importance of bacterial communities, much of the research over the past one hundred years has focused on the growth and physiology of microbes grown in pure culture (Stubbendieck et al., 2016). Only recently, thanks in part to the availability of novel experimental and computational tools, the dynamics and ecological significance of bacterial communities have started to be systematically studied (Czaran et al., 2002; Kerr et al., 2002; Reichenbach et al., 2007; Kim et al., 2008; Pérez-Gutiérrez et al., 2013; Blanchard and Lu, 2015; Hol et al., 2015; Cordero and Datta, 2016; Stubbendieck and Straight, 2016; Stubbendieck et al., 2016). Two different questions in particular have received attention from an ecological perspective: (i) how bacterial interactions at the microscale level drive the emergence of spatial colony structures (Kerr et al., 2002; Kim et al., 2008; Pérez-Gutiérrez et al., 2013; Blanchard and Lu, 2015; Hol et al., 2015; Tekwa et al., 2015; Cordero and Datta, 2016; Wilmoth et al., 2018), and (ii) how these interactions (mainly antagonistic ones) promote microbial biodiversity (Czaran et al., 2002; Kerr et al., 2002; Reichenbach et al., 2007; Narisawa et al., 2008; Pérez-Gutiérrez et al., 2013).

In a recent work, Pérez-Gutiérrez et al. (2013) characterized the antagonistic interactions of a set of culturable thermoresistant bacteria isolated from the Churince lagoon in Cuatro Cienegas, Mexico (Cerritos et al., 2011). This lagoon constitutes a still and homogeneous water environment with physicochemical conditions that are stable and constant throughout the year. Pérez-Gutiérrez et al. found that the community structure at sampling sites separated by tens of meters varies greatly and that there is a strong presence of antagonistic bacteria that makes this a hierarchical, aggressive, food web-like community, with four types of behaviors: (a) resistant bacteria that are not affected by other bacteria, (b) top antagonistic bacteria that antagonize other bacteria and are not antagonized by any, (c) intermediate antagonistic bacteria that are antagonized and also antagonize others, and (d) sensitive bacteria that are antagonized by and do not antagonize other bacteria. These findings disagree with the long standing hypothesis of microbial ecology stating that “everything is everywhere, the system selects” (Bass-Becking, 1934; O'Malley, 2007). On the contrary, the results from Pérez-Gutiérrez et al. suggest that biotic factors in the form of antagonistic interactions shape the community structure at the different sampling sites. This conclusion was further supported by Zapién-Campos et al. (2015), who simulated, by means of a cellular-automata model, the evolution of the bacterial strains studied by Pérez-Gutiérrez et al. (2013), taking into account the reported matrix of antagonistic interactions. The results of Zapién-Campos et al. suggest that resistant bacteria can promote co-existence of all bacterial populations and that they do this by limiting direct physical interaction between antagonist and sensitive bacteria. They also simulated the action of water flow by randomly shuffling the position of all bacteria and observed that the protective effect was lost and sensitive bacteria perish, meaning that spatial structure is necessary to maintain co-existence.

The present work is aimed at testing the Zapién-Campos et al. hypothesis that resistant bacterial strains protect sensitive ones from antagonistic bacteria by creating physical barriers. To this end, we selected three bacterial strains from the set isolated and studied by Pérez-Gutiérrez et al. (2013): one antagonistic, one sensitive, and one resistant, and implemented experimental protocols to quantitatively study the effects of interactions among them on the dynamics and spatial structure of mixed plate cultures. We must point out that very little is known about these bacterial strains as they were only recently isolated from the wild. Indeed, this is the first time they are studied beyond their antagonistic interactions. Our experimental results suggest a dynamic protective mechanism conferred by the resistant strain, which is different to that hypothesized by Zapién-Campos et al. The feasibility of this mechanism was further tested by means of a simple *ad hoc* computational model.

## 2. Materials and Experimental Methods

### 2.1. Bacterial Strains and Growth Conditions

The collection of 78 thermoresistant bacterial strains, isolated from the Churince lagoon in Cuatro Cienegas, Mexico, and studied by Pérez-Gutiérrez et al. (2013), was kindly donated by Prof. Gabriela Olmedo, from the Department of Genetic Engineering at Cinvestav, Mexico. All 78 strains are maintained at –80°C in marine broth (sodium chloride 5 g/L, magnesium sulfate 4.8 g/L, sodium sulfate 1 g/L, calcium chloride 0.4 g/L, potassium chloride 0.2 g/L, ferric citrate 0.1 g/L, sodium carbonate 0.1 g/L, potassium bromide 0.08 g/L, dibasic sodium phosphate 0.08 g/L, sodium fluoride 0.024 g/L, boric acid 0.022 g/L, ammonium nitrate 0.016 g/L, peptone 5 g/L, yeast extract 1 g/L) with 15% glycerol.

Three strains were selected from the collection: one antagonistic (*Bacillus pumilus*), one sensitive (*Bacillus aquimaris*), and one resistant (*Staphylococcus* sp.). To perform the experiments, bacteria were collected from stock, plated on a Petri dish with marine broth plus 2% agar, and incubated overnight at 28°C (For *OD*_600_ see “Bacterial-community agar-plate experiments” subsection below). A single colony was then selected to inoculate 1 mL of marine broth and incubated again at 28°C overnight. Finally, at least, 100 μL of this last culture were used to inoculate 5–10 mL of fresh marine broth, and the resulting suspension was incubated at 28°C prior to experimentation. We made sure that the incubation time of the last step was such that the bacterial populations were at some point of their exponential growth phase.

### 2.2. Antagonism Assays

Antagonistic relations were confirmed by means of the spot-lawn assay (Burkholder et al., 1966). A layer of warm liquid marine medium with 0.45% agar, inoculated with 5% of a given bacterial strain culture (For *OD*_600_ see “Bacterial-community agar-plate experiments” subsection below), was poured on a Petri dish with marine broth plus 2% agar and let to dry. Later, drops of 5 μL of another strain culture were placed on top of the lawn. The plate was then incubated at 28°C overnight.

### 2.3. Neighboring-Colony Dynamics Experiments

To quantify the expansion of a colony in the vicinity of another colony, 1 μL bacterial-culture drops were inoculated on a Petri dish with a separation of 15 mm. Petri dishes were incubated at 28°C with 65% relative humidity to avoid agar drying. Digital images were recorded daily (for 6 days) from closed Petri dishes by means of a scanner (Epson perfection 1650, 600 dpis). Recorded images were analyzed with the Fiji distribution of the ImageJ software (Schindelin et al., 2012).

### 2.4. Bacterial-Community Agar-Plate Experiments

Cell concentration in bacterial cultures was estimated by measuring the corresponding optical densities at 600 nm (*OD*_600_). This measurement was calibrated for each strain using the count of colony-forming units (cfus) as a direct measurement. The *OD*_600_ measurements were 1.0 ± 0.1, 0.8 ± 0.1, and 1.6 ± 0.2 for the sensitive, antagonist, and resistant strains respectively. The correspondent concentrations, in the same order as before, were 1.0e+08, 5.6e+08, and 2.4e+09 cfus/mL.

Artificial communities were created by mixing a given amount of sensitive bacteria, plus different proportions of antagonistic bacteria (10, 25, 50, 75, 100, 200%), plus (in some cases) 500% of resistant bacteria. The mixture was homogenized by gently pipetting. Five microliters drops were inoculated on a plate of marine media plus 2% agar, and incubated at 28°C for 4 days and 65% relative humidity. Digital images were recorded from closed plates by means of a scanner, and the area fraction corresponding by each strain was measured via image analysis with ImageJ.

## 3. Experimental Results

**Figure 1:**
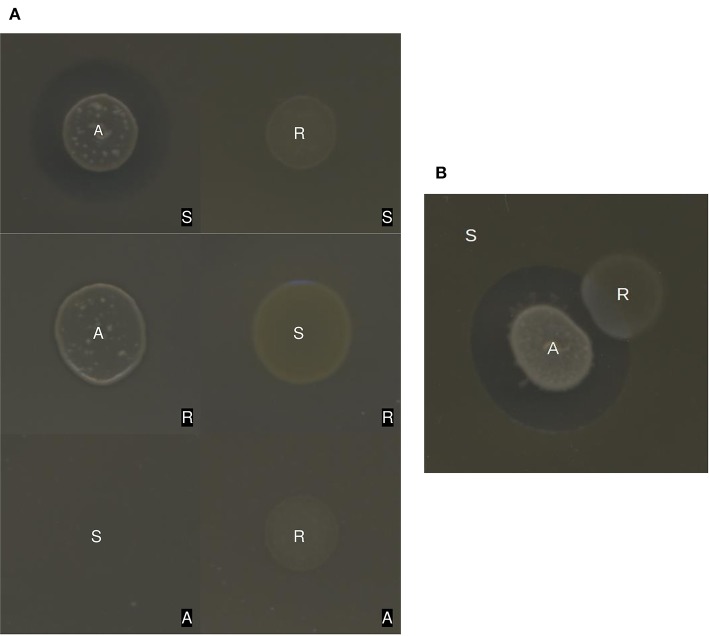
**(A)** Spot-lawn antagonism assays for the antagonistic (A), sensitive (S), and resistant (R) bacteria. The letters on the center of each colony indicate the spots, while the letters in black boxes indicate the lawns. A antagonizes S (upper left and lower left), R can grow with A and S (everything else). **(B)** Antagonism assay in which A and R are inoculated close together on top of a S lawn. The presence of R does not affect the inhibition halo.

We started by selecting three different strains from the collection isolated and studied by Pérez-Gutiérrez et al. (2013): one antagonistic, one sensitive, and one resistant. We took care of picking them so their colonies could be easily told apart in plain sight. This is necessary for differentiating the patches corresponding to different strains when they are co-cultured on a Petri dish. The bacterial strains thus chosen were *Bacillus pumilus* (antagonistic), *Bacillus aquimaris* (sensitive), and *Staphylococcus* sp. (resistant). We performed the corresponding antagonism assays to discard the possibility that the selected bacterial strains have suffered any modification that altered their antagonistic interactions. The results are presented in Figure 1A. We can see there that all three strains behave as expected. That is, the antagonistic-strain colony creates an inhibition halo where sensitive bacteria cannot grow, a lawn of antagonistic bacteria wipes out the sensitive colony, and no interactions are observed between the antagonistic and the resistant strains, as well as between the sensitive and the resistant strains.

We further inoculated the antagonistic and the resistant strains close together, on top of the sensitive lawn. The result of this experiment is illustrated in Figure 1B. Observe that the extent and the effect of the antagonistic inhibition halo is not affected by the presence of a resistant colony. This means that, contrary to the assumptions in the model by Zapién-Campos et al. (2015), the resistant strain cannot physically isolate a sensitive colony from the diffusible compound presumably secreted by antagonistic bacteria.

The results illustrated in Figure 1B indicate that the protective mechanism suggested by Zapién-Campos et al. does not take place in the selected set of bacterial strains. This could mean that the resistant strain does not affect the population dynamics of antagonistic and sensitive strains, when the three of them are co-cultured on a Petri dish. To test whether this is the case, we created artificial bacterial communities by mixing a given amount of sensitive bacteria, plus five times as much resistant bacteria, plus variable amounts of antagonistic bacteria, inoculated them on Petri dishes, and let them grow for 4 days. The results of these experiments are contrasted with those of control experiments with artificial mixed colonies containing the sensitive and antagonistic strains only.

**Figure 2:**
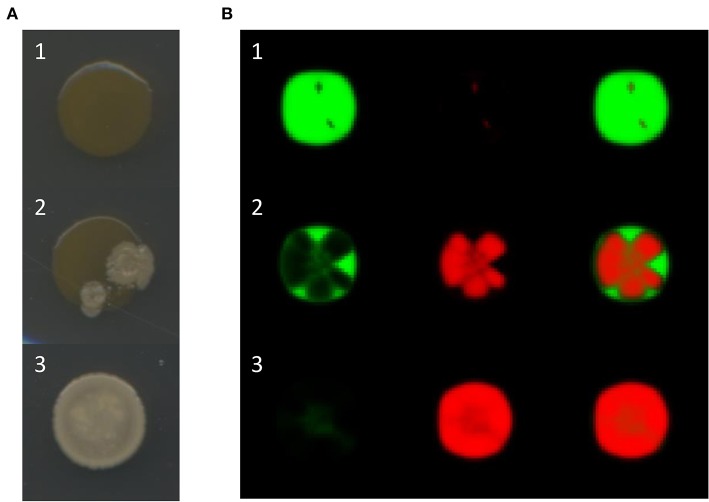
**(A)** Observed outcomes of artificial bacterial communities growing as a single colony. Either the final morphology of the mixed colony is that of the sensitive strain *B. aquimaris* (A1), that of the antagonistic strain *B. pumilus* (A3), or a combination of both (A2). **(B)** Simulation results for mixed colonies with different outcomes: row B1, the sensitive strains dominates; row B2, the mixed colony presents patches in which either the sensitive or the antagonistic strains dominate; row B3, the antagonistic strain dominates. The difference between these simulations is the initial proportion of antagonistic bacteria: 0.1, 0.3, and 1.0 for rows B1, B2, and B3, respectively. The images in the left and center columns represent the distributions of the sensitive and antagonistic strains, respectively, while the images in the right column show both populations.

The characteristic outcomes observed in the experiments described in the former paragraph are illustrated in Figure 2A. Observe that in some cases, the final morphology of the mixed colony corresponds to either that of the sensitive or the antagonistic strains, while in other cases a combination of both morphologies can be appreciated. Interestingly, the morphology of the resistant strain is never observed. To better understand this phenomenon, we mixed sensitive and resistant bacteria cultures and plated the mixed culture on Petri dishes. In all cases we observed that, no matter what the initial amount of resistant bacteria was, the mixed colony always acquired the morphology of the sensitive strain. However, when samples of the resulting colonies were diluted and spread on Petri dishes, colonies of both bacterial strains appeared. Thus, it seems that resistant and sensitive bacteria co-exist, even though the morphology of the first one is not apparent in the mixed colonies.

**Figure 3:**
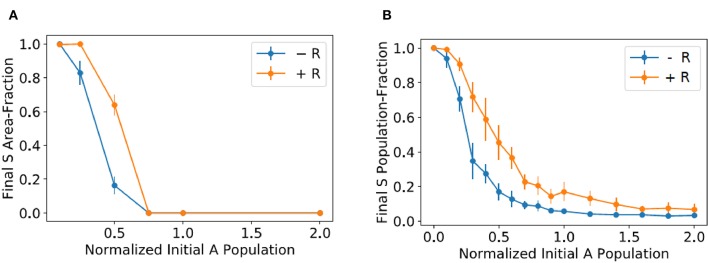
**(A)** Average mixed-colony area fraction corresponding the morphology of the sensitive strain vs. the amount of antagonistic bacteria in the initial mixture (normalized to the initial amount of sensitive bacteria). Blue dots correspond to mixed colonies with no resistant strain, while a number of resistant bacteria equal to five times the initial amount of sensitive ones was added in the experiments represented by orange dots. **(B)** Plots of the average fraction of sensitive bacteria in mixed-colony simulations vs. the initial amount of antagonistic bacteria, normalized to the initial sensitive population.

To quantify the experiments illustrated in Figure 2A, we measured in every mixed colony the area fraction corresponding to the morphology of the sensitive strain and averaged over all the mixed colonies with the same conditions (amounts of added resistant and antagonistic bacteria). We performed two independent experiments, with 10 colonies in each experiment, for every experimental condition. The results are reported in Figure 3A. Observe that the sensitive area-fraction decreases in a sigmoidal fashion as the amount of added antagonistic bacteria increases. The same behavior is observed when resistant bacteria are included in the initial mixture, but the whole curve is displaced to the right. This demonstrates that the resistant strain confers some kind of protection to sensitive bacteria. However, as previously discussed, the underlying mechanism cannot be that of spatial isolation.

**Figure 4:**
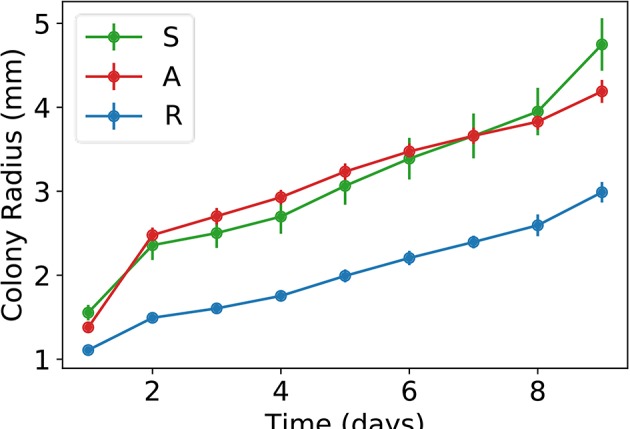
Plots of average colony radius vs. time for the three different strains here studied: S, sensitive; A, antagonistic; and R, resistant. Bars corresponds to standard deviations.

To improve our understanding of the interactions between the sensitive, antagonistic, and resistant strains, we performed a series of dynamic experiments. First of all, we inoculated single colonies on Petri dishes and measured the time evolution of their radii. The results are shown in Figure 4. Notice that the growth rates of the antagonistic and sensitive strains are very similar, while the resistant strain grows at a noticeably smaller rate, which explains the absence of resistant patches in the community colonies.

**Figure 5:**
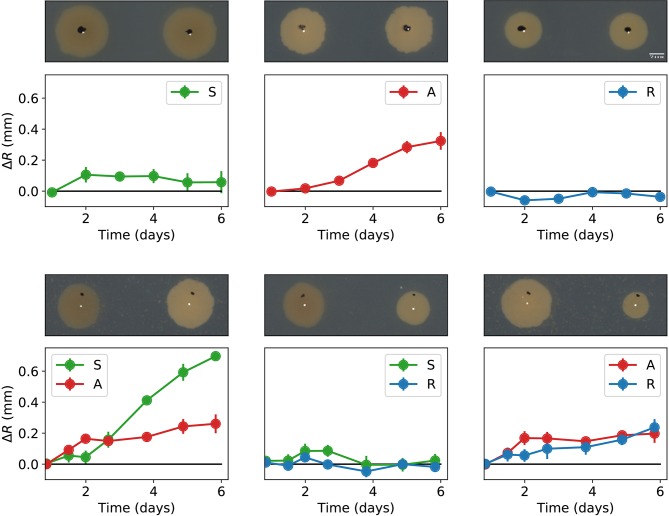
Pair-wise interactions between all combinations of the antagonist (A), sensitive (S), and resistant (R) bacterial colonies inoculated with a separation of 15 mm. The graphs in the top row correspond to colonies of the same strain facing each other, while the graphs in the bottom row correspond to colonies of different strains. The plots in each graph indicate the time evolution of Δ*R*. Notice that A always has a slower internal radius independently of the type of neighbor colony. Error bars denote standard errors. A representative picture of the corresponding experiment is shown on top of every plot. The black dots in these pictures are alignment marks made on the Petri dishes, while the white dots are the calculated centers after 1 day of growth.

We also carried out experiments in which two different colonies were inoculated on a Petri dish with a separation of 15 mm, and their external (in the direction opposite to the other colony) and internal (in the direction of the other colony) radii were periodically measured for 6 days. Let Δ*R* denote the difference between the external and internal radii of a colony at a given time. From its definition, Δ*R* > 0 means decremented growth in the direction of the other inoculated colony. The obtained Δ*R* values (averaged over four independent experiments) are reported in Figure 5. Observe that whenever a colony of the antagonistic strain is inoculated in the proximity of another colony (either sensitive, resistant, or antogonistic), there is a decrease of its internal radius as compared to the external one. The colonies that are inoculated close to the antagonistic strain also grow more slowly in their internal direction. However, the growth decrease is about three times as large for the sensitive colony than it is for the resistant one. Finally, when the sensitive and the resistant strains are inoculated close to each other, not much difference between their internal and external radii is observed.

One way to interpret the results in the previous paragraph is that antagonistic bacteria are capable of detecting the proximity of other cells. When this happens, the former bacteria respond by secreting a substance meant to attack neighboring bacteria. This however conveys a metabolic cost, which implies a reduction of the antagonistic bacteria growth rate. On the other hand, the growth rate of sensitive bacteria is largely affected by the antagonistic substance, whereas that of resistant bacteria is not affected as much. This could happen because resistant bacteria pay a metabolic cost to resist the antagonistic substance. To test whether these interactions are enough to explain the dynamic behavior represented in Figure 3A, we developed a coupled-map lattice model, which we introduce and analyze in the following section.

## 4. Mathematical Model Development and Simulation Results

In the present model, the surface where the colony grows is represented as a regular square lattice. Each lattice compartment corresponds to a small area where all bacteria populations may co-exist. Let *s*_*i, j*_ and *a*_*i, j*_ respectively represent the normalized populations (normalized to the corresponding carrying capacity) of sensitive and antagonistic bacteria in the compartment with coordinates (*i, j*). Under the assumption that both species interact within the compartment according to the Lotka-Volterra competition model (Bomze, 1983), and that the growth rate of sensitive bacteria is negatively affected by the antagonistic cells present in their neighborhood, the amount of bacteria that reproduce in a single model iteration is

(1) $$\begin{array}{rcl} \Delta s_{i,j}^{+} & = & \frac{r_{s}}{1+{\left(Na_{i,j}/K_{a}\right)}^{4}}\left(1-s_{i,j}-\alpha a_{i,j}\right)s_{i,j}, \end{array}$$

(2) $$\begin{array}{rcl} \Delta a_{i,j}^{+} & = & r_{a}\left(1-a_{i,j}-\beta s_{i,j}\right)a_{i,j}, \end{array}$$

where

(3) $$\begin{array}{l} Na_{i,j}=a_{i,j}/2\\ \;+\left(a_{i,j-1}+a_{i,j+1}+a_{i-1,j}+a_{i,j+1}\right)/12\\ \;+\left(a_{i-1,j-1}+a_{i-1,j+1}+a_{i+1,j-1}+a_{i+1,j+1}\right)/24. \end{array}$$

In the equations above, *r*_*s*_ and *r*_*a*_ respectively represent the intrinsic growth rates of the sensitive and antagonistic strains; α and β denote the level of competition between both strains; and *K*_*a*_ stands for the sensitivity of the sensitive-strain growth rate to the presence of neighboring antagonistic bacteria.

The model also assumes that bacteria of both populations diffuse out in the following amounts during one iteration:

(4) $$\begin{array}{rcl} \Delta s_{i,j}^{-} & = & D_{s}s, \end{array}$$

(5) $$\begin{array}{rcl} \Delta a_{i,j}^{-} & = & D_{a}a, \end{array}$$

and that they distribute among the neighboring compartments as follows:

$$\begin{array}{l} \delta s_{\left\{i,j\right\}\to \left\{i-1,j\right\}}^{+}=\delta s_{\left\{i,j\right\}\to \left\{i+1,j\right\}}^{+}=\delta s_{\left\{i,j\right\}\to \left\{i,j-1\right\}}^{+}\\ \;=\delta s_{\left\{i,j\right\}\to \left\{i,j+1\right\}}^{+}=\Delta s_{i,j}^{-}/6,\\ \delta s_{\left\{i,j\right\}\to \left\{i-1,j-1\right\}}^{+}=\delta s_{\left\{i,j\right\}\to \left\{i-1,j+1\right\}}^{+}=\delta s_{\left\{i,j\right\}\to \left\{i+1,j-1\right\}}^{+}\\ \;=\delta s_{\left\{i,j\right\}\to \left\{i+1,j+1\right\}}^{+}=\Delta s_{i,j}^{-}/12,\\ \delta a_{\left\{i,j\right\}\to \left\{i-1,j\right\}}^{+}=\delta a_{\left\{i,j\right\}\to \left\{i+1,j\right\}}^{+}=\delta a_{\left\{i,j\right\}\to \left\{i,j-1\right\}}^{+}\\ \;=\delta a_{\left\{i,j\right\}\to \left\{i,j+1\right\}}^{+}=\Delta a_{i,j}^{-}/6,\\ \delta a_{\left\{i,j\right\}\to \left\{i-1,j-1\right\}}^{+}=\delta a_{\left\{i,j\right\}\to \left\{i-1,j+1\right\}}^{+}=\delta a_{\left\{i,j\right\}\to \left\{i+1,j-1\right\}}^{+}\\ \;=\delta a_{\left\{i,j\right\}\to \left\{i+1,j+1\right\}}^{+}=\Delta a_{i,j}^{-}/12. \end{array}$$

From the above considerations, the evolution of the lattice model is simulated by means of an algorithm implemented in Python, whose pseudocode is as follows:

1. Set the initial values of variables *s*_*i, j*_ and *a*_*i, j*_ for all the lattice compartments.
2. For every lattice compartment compute $\Delta s_{i,j}^{+}$, $\Delta a_{i,j}^{+}$, $\Delta s_{i,j}^{-}$, $\Delta a_{i,j}^{-}$, $\delta s_{\left\{i,j\right\}\to \left\{i^{\prime },j^{\prime }\right\}}^{+}$, and $\delta a_{\left\{i,j\right\}\to \left\{i^{\prime },j^{\prime }\right\}}^{+}$.
3. Compute the new values for *s*_*i, j*_ and *a*_*i, j*_ as follows:
  $$\begin{array}{r} s_{i,j}+=\Delta s_{i,j}^{+}-\Delta s_{i,j}^{-}+\underset{i^{\prime },j^{\prime }}{\sum }s_{\left\{i^{\prime },j^{\prime }\right\}\to \left\{i,j\right\}}^{+},\\ a_{i,j}+=\Delta a_{i,j}^{+}-\Delta a_{i,j}^{-}+\underset{i^{\prime },j^{\prime }}{\sum }a_{\left\{i^{\prime },j^{\prime }\right\}\to \left\{i,j\right\}}^{+}, \end{array}$$
  where *i*′ = *i* − 1, *i, i* + 1 and *j*′ = *j* − 1, *j, j* + 1.
4. Iterate to 2.

The present model can be seen as an approximated implementation of Euler's algorithm to solve the differential equations describing Lotka-Volterra competition within a compartment, as well as diffusion among lattice compartments. In this regard, parameters *r*_*s*_ and *r*_*a*_ would correspond to population growth rates, multiplied by Δ*t* (the time period corresponding to one algorithm iteration). Similarly, *D*_*s*_ and *D*_*a*_ would correspond to the diffusion rates times Δ*t*. Given that the smaller the value of Δ*t* the better approximated the solutions yielded by Euler's algorithm, it follows that *r*_*s*_, *r*_*a*_, *D*_*s*_, *D*_*a*_≪1 in order for our model to accurately simulate Lotka-Volterra competition and diffusion. On the other hand, since we are interested in qualitatively understanding the evolution of mixed colonies, we do not need to know the specific values of such parameters, but only how they compare among each other.

If initially, the values of *s*_*i, j*_ are set equal to 1 in the lattice compartments lying within a small circle and 0 elsewhere, whereas *a*_*i, j*_ = 0 everywhere, the model simulates a more or less circular colony whose radius grows linearly with time, with a speed that is proportional to the product *r*_*s*_*D*_*s*_ (results not shown). On the other hand, setting *D*_*s*_ ≪ *r*_*s*_ ensures that *s*_*i, j*_ ≈ 1 almost everywhere in the colony, except for the bordering compartments. From these considerations and taking into account that sensitive and antagonistic colonies grow at similar rates—see Figure 4, we set:

(6) $$\begin{array}{r} r_{s}=r_{a}=0.1,\;D_{s}=D_{a}=0.01. \end{array}$$

On the other hand, parameter *K*_*a*_ is set to

(7) $$\begin{array}{r} K_{a}=0.08. \end{array}$$

The reason for this choice is explained below.

We initialized the lattice model as follows to simulate mixed colony experiments. The lattice size was set to 51 × 51, with periodic boundary conditions. After identifying all the lattice compartments within a radius of 5 compartments from the lattice center, the following initial values for variables *s*_*i, j*_ and *u*_*i*, 1_ were set:

$$\begin{array}{l} s_{i,j}^{o}=\left\{ \begin{array}{l} \xi ,\text{​ within the circle}\\ 0,\text{​ outside} \end{array}\right.\\ a_{i,j}^{o}=\left\{ \begin{array}{l} \varsigma ,\text{​ within the circle}\\ 0,\text{​ outside} \end{array}\right. \end{array}$$

where ξ and ζ are uniformly distributed random variables. ξ takes values in the interval [0, 0.2], while ζ does it in the interval [0, 0.2ρ], with rho being the proportion of antagonistic cells in the initial mixture. This way of initializing the lattice emulates a diluted initial colony, with the corresponding heterogeneous distribution of bacteria due to their low initial numbers.

After initializing the lattice, we employed the algorithm previously described to simulate the evolution of mixed colonies of sensitive and antagonistic bacteria. We show in Figure 2B the results of three different simulations in which the algorithm was run for 200 iterations. Notice how, by modifying the initial proportion of antagonistic bacteria, we obtained outcomes that qualitatively resemble those of the mixed colony experiments.

To compare with the experimental results in Figure 3A, we computed the total populations of sensitive and antagonistic bacteria at the end of several simulations:

$$S=\underset{i,j=1\ldots 51}{\sum }s_{i,j},\;A=\underset{i,j=1\ldots 51}{\sum }a_{i,j},$$

and calculated the fraction of sensitive bacteria as:

$$\sigma =\frac{S}{S+A}.$$

We performed 10 independent simulation for every initial proportion of antagonistic bacteria and then computed the final average fraction of sensitive bacteria. In these simulations, we took the parameter values reported in Equation (6) and considered many different *K*_*a*_ values. We found by trial and error that by setting *K*_*a*_ to the value in Equation (7) the model renders results—see Figure 3B—that agree with those in Figure 3A, in which resistant bacteria are absent in the mixed colony.

To model the effect of adding resistant bacteria to the mixed colony, while keeping the model as simple as possible, we took into account that, according to the results in Figure 2B, antagonistic bacteria reduce their growth rate whenever they are close to other cells. Moreover, if we consider that resistant bacteria are five times more numerous than sensitive bacteria in the initial colonies, one can assume that they are more or less homogeneously distributed, and that they surround all antagonistic and sensitive bacteria. Thus, since the resistant strain does not affect the growth of sensitive bacteria, we could assume that the effect of adding it to the initial mixture is to decrease the growth rate of all antagonistic bacteria. Based on the above considerations, we repeated the last simulations by decreasing the value or *r*_*a*_ from 0.1 to 0.09, and plotted the results in Figure 3B. Notice that the simulation predictions qualitatively agree with the results in Figure 3A corresponding to the experiments in which the mixed colony includes resistant bacteria.

## 5. Concluding Remarks

We have studied the population dynamics of Petri-dish mixed colonies, consisting of three different bacterial strains selected from the collection of culturable, thermoresistant bacteria originally isolated (from the Churince lagoon in Cuatro Cienegas, Mexico) and studied by Pérez-Gutiérrez et al. (2013). The present work was directly motivated by Pérez-Gutiérrez et al. (2013) and Zapién-Campos et al. (2015). In particular, our objective was to test the hypothesis by Zapién-Campos et al. (2015) that the co-existence of antagonistic and sensitive strains is due to resistant colonies that form physical barriers which impede antagonistic bacteria to attack sensitive ones.

We chose three different bacterial strains from the Pérez-Gutiérrez et al. collection: one antagonistic, *B. pumilus*; one sensitive, *B. aquimaris*; and one resistant, *Staphylococcus* sp. We were able to prove with these strains that, although the protective mechanism suggested by Zapién-Campos et al. (2015) does not work in this case, the resistant strain confers some kind of protection to sensitive bacteria when the three of them are present in mixed colonies.

Further experimental results showed that *B. pumilus* colonies slightly decrease their growth rate in the proximity of *B. aquimaris, Staphylococcus* sp. or even another *B. pumilus* colony. We speculate that this is due to the metabolic cost implied in the detection of adjacent colonies or the production and secretion of an antagonistic substance, in response to the presence of potentially competing bacteria. Our experiments also demonstrated that the growth rate of sensitive colonies shows a large decrease in the proximity of antagonistic colonies, while the growth rate of resistant colonies is also affected but not to the same extent. These results, together with those of the antagonism assays, could mean that resistant bacteria respond by activating some inherent mechanism to resist the antagonistic substance (with the corresponding metabolic cost), while sensitive bacteria are directly affected by this substance.

We developed a coupled-map lattice model to verify whether these mechanisms might explain the observed protective effect conferred by resistant upon sensitive bacteria, and we found that this is indeed possible. Hence, our modeling and experimental results suggest that the presence of resistant bacteria indirectly improves the probability that patches of sensitive bacteria grow in a mixed colony. By making antagonistic bacteria produce and secrete an antagonistic substance (with the concomitant metabolic cost and growth rate reduction), resistant bacteria increase the likelihood that sensitive bacteria locally outcompete antagonistic ones.

The metabolic cost for producing antagonistic or resistance compounds, and its association with a growth penalty, has been previously studied experimentally and theoretically in other systems (Kerr et al., 2002; Conlin et al., 2014; Aguirre-von Wobeser et al., 2015). If the resistant organisms pay a lower cost than the antagonistic ones, while sensitive individuals have the greatest growth rate in the community, a rock-paper-scissor dynamics arises (Pagie and Hogeweg, 1999; Frean and Abraham, 2001). Even though biotic interactions in our system have the same topology, rock-paper-scissor dynamics is not present because *Staphylococcus* sp. (the resistant strain) cannot outcompete *B pumilus* (the antagonistic strain). In our system, the advantage of antagonistic bacteria when competing with sensitive strains is not questionable. However, this advantage is attenuated with the addition of a resistant strain. This attenuation may be due to an increased penalty for responding to multiple neighboring strains, which prevent the antagonist from growing and expanding. If we naively attempt to extrapolate these findings to the scale of a natural bacterial community with hundreds of bacterial strains, the described antagonistic strategy may not be very competitive. However, further studies with larger communities are required to grasp the complexity of this and other types of interactions.

From a different perspective, the fact that in the present work we have studied a bacterial community with an interaction network (antagonist-sensitive-resistant) that has been tackled elsewhere, may be seen as a weakness at first sight. However, it is not because our results reveal that this network can show more complex and subtle behaviors, which perhaps cannot be extrapolated to the scale of natural microbial communities, but which represent a basis for future studies with larger communities.

Finally, we acknowledge that a chemical characterization of the interactions between the bacterial populations studied here is required for a deeper understanding of the system behavior. In this respect, we wish to point out that such bacterial strains were recently isolated from the wild, and thus many aspects about their behavior are still unknown. As a matter of fact, this is the first report in which they are studied beyond their antagonistic interactions. Moreover, the methodologies needed to investigate the nature of their chemical interactions are beyond the scope of the present study, and hence we leave this as an open problem for future work.

## Data Availability Statement

The raw data supporting the conclusions of this manuscript will be made available by the authors, without undue reservation, to any qualified researcher.

## Author Contributions

OG-N performed research, contributed analytic tools, analyzed data, and contributed to writing the manuscript. MS designed research, contributed analytic tools, performed research, analyzed data, and wrote the manuscript. All authors reviewed the manuscript.

### Conflict of Interest

The authors declare that the research was conducted in the absence of any commercial or financial relationships that could be construed as a potential conflict of interest.

## References

[B1] Aguirre-von Wobeser E. Eguiarte L. E. Souza V. Soberón-Chávez G. (2015). Theoretical analysis of the cost of antagonistic activity for aquatic bacteria in oligotrophic environments. Front. Microbiol. 6:490. 10.3389/fmicb.2015.00490 26074891 PMC4444843

[B2] Anand A. A. P. Sripathi K. (2004). Digestion of cellulose and xylan by symbiotic bacteria in the intestine of the indian flying fox (*Pteropus giganteus*). Comp. Biochem. Physiol. A Mol. Integr. Physiol. 139, 65–69. 10.1016/j.cbpb.2004.07.006 15471682

[B3] Bass-Becking L. G. M. (1934). Geobiologie of Inleiding tot de Milieukunde. Diligentia-voordrachten. The Hague: W.P. Van Stockum & Zoon.

[B4] Blanchard A. E. Lu T. (2015). Bacterial social interactions drive the emergence of differential spatial colony structures. BMC Syst. Biol. 9:59. 10.1186/s12918-015-0188-5 26377684 PMC4573487

[B5] Bomze I. M. (1983). Lotka-volterra equation and replicator dynamics: a two-dimensional classification. Biol. Cybernet. 48, 201–211. 10.1007/BF00318088

[B6] Burkholder P. R. Pfister R. M. Leitz F. H. (1966). Production of a pyrrole antibiotic by a marine bacterium. Appl. Microbiol. 14, 649–653. 4380876 10.1128/am.14.4.649-653.1966 PMC546803

[B7] Cerritos R. Eguiarte L. E. Avitia M. Siefert J. Travisano M. Rodríguez-Verdugo A.. (2011). Diversity of culturable thermo-resistant aquatic bacteria along an environmental gradient in cuatro ciénegas, coahuila, méxico. Antonie Van Leeuwenhoek 99, 303–318. 10.1007/s10482-010-9490-9 20711674

[B8] Conlin P. L. Chandler J. R. Kerr B. (2014). Games of life and death: antibiotic resistance and production through the lens of evolutionary game theory. Curr. Opin. Microbiol. 21:35–44. 10.1016/j.mib.2014.09.004 25271120

[B9] Cordero O. X. Datta M. S. (2016). Microbial interactions and community assembly at microscales. Curr. Opin. Microbiol. 31, 227–234. 10.1016/j.mib.2016.03.015 27232202 PMC5157693

[B10] Currie C. R. (2001). A community of ants, fungi, and bacteria: a multilateral approach to studying symbiosis. Annu. Rev. Microbiol. 55, 357–380. 10.1146/annurev.micro.55.1.357 11544360

[B11] Czaran T. L. Hoekstra R. F. Pagie L. (2002). Chemical warfare between microbes promotes biodiversity. Proc. Natl. Acad. Sci. U.S.A. 99, 786–790. 10.1073/pnas.012399899 11792831 PMC117383

[B12] Falkowski P. G. Fenchel T. Delong E. F. (2008). The microbial engines that drive earth's biogeochemical cycles. Science 320, 1034–1039. 10.1126/science.1153213 18497287

[B13] Fisher R. F. Long S. R. (1992). Rhizobium–plant signal exchange. Nature 357:655. 10.1038/357655a0 1614514

[B14] Frean M. Abraham E. R. (2001). Rock–scissors–paper and the survival of the weakest. Proc. R. Soc. Lond. Ser B Biol. Sci. 268, 1323–1327. 10.1098/rspb.2001.1670 11429130 PMC1088744

[B15] Hol F. J. H. Galajda P. Woolthuis R. G. Dekker C. Keymer J. E. (2015). The idiosyncrasy of spatial structure in bacterial competition. BMC Res. Notes 8:245. 10.1186/s13104-015-1169-x 26081497 PMC4470050

[B16] Kerr B. Riley M. A. Feldman M. W. Bohannan B. J. M. (2002). Local dispersal promotes biodiversity in a real-life game of rock–paper–scissors. Nature 418, 171–174. 10.1038/nature00823 12110887

[B17] Kim H. J. Boedicker J. Q. Choi J. W. Ismagilov R. F. (2008). Defined spatial structure stabilizes a synthetic multispecies bacterial community. Proc. Natl. Acad. Sci. U.S.A. 105, 18188–18193. 10.1073/pnas.0807935105 19011107 PMC2587551

[B18] Narisawa N. Haruta S. Arai H. Ishii M. Igarashi Y. (2008). Coexistence of antibiotic-producing and antibiotic-sensitive bacteria in biofilms is mediated by resistant bacteria. Appl. Environ. Microbiol. 74, 3887–3894. 10.1128/AEM.02497-07 18441106 PMC2446560

[B19] Ofosu A. (2016). *Clostridium difficile* infection: a review of current and emerging therapies. Ann. Gastroenterol. 29, 147–154. 10.20524/aog.2016.0006 27065726 PMC4805733

[B20] Ohno S. (1997). The reason for as well as the consequence of the cambrian explosion in animal evolution. J. Mol. Evol. 44, S23–S27. 10.1007/PL00000055 9071008

[B21] O'Malley M. A. (2007). The nineteenth century roots of 'everything is everywhere'. Nat. Rev. Microbiol. 5, 647–651. 10.1038/nrmicro1711 17603517

[B22] Pagie L. Hogeweg P. (1999). Colicin diversity: a result of eco-evolutionary dynamics. J. Theor. Biol. 196, 251–261. 10.1006/jtbi.1998.0838 10049618

[B23] Pérez-Gutiérrez R.-A. López-Ramírez V. Islas Á. Alcaraz L. D. Hernández-González I. Olivera B. C. L.. (2013). Antagonism influences assembly of a bacillus guild in a local community and is depicted as a food-chain network. ISME J. 7, 487–497. 10.1038/ismej.2012.119 23096405 PMC3578566

[B24] Petrof E. O. Gloor G. B. Vanner S. J. Weese S. J. Carter D. Daigneault M. C.. (2013). Stool substitute transplant therapy for the eradication of clostridium difficile infection: ‘RePOOPulating' the gut. Microbiome 1:3. 10.1186/2049-2618-1-3 24467987 PMC3869191

[B25] Reichenbach T. Mobilia M. Frey E. (2007). Mobility promotes and jeopardizes biodiversity in rock–paper–scissors games. Nature 448, 1046–1049. 10.1038/nature06095 17728757

[B26] Schindelin J. Arganda-Carreras I. Frise E. Kaynig V. Longair M. Pietzsch T. (2012). Fiji: an open-source platform for biological-image analysis. Nat. Methods 28, 676–682. 10.1038/nmeth.2019 PMC3855844 22743772

[B27] Schopf J. W. (1993). Microfossils of the early archean apex chert: new evidence of the antiquity of life. Science 260, 640–646. 10.1126/science.260.5108.640 11539831

[B28] Stubbendieck R. M. Straight P. D. (2016). Multifaceted interfaces of bacterial competition. J. Bacteriol. 198, 2145–2155. 10.1128/JB.00275-16 27246570 PMC4966439

[B29] Stubbendieck R. M. Vargas-Bautista C. Straight P. D. (2016). Bacterial communities: interactions to scale. Front. Microbiol. 7:1234. 10.3389/fmicb.2016.01234 27551280 PMC4976088

[B30] Tekwa E. W. Nguyen D. Juncker D. Loreau M. Gonzalez A. (2015). Patchiness in a microhabitat chip affects evolutionary dynamics of bacterial cooperation. Lab Chip 15, 3723–3729. 10.1039/C5LC00576K 26224163

[B31] Turnbaugh P. J. Ley R. E. Hamady M. Fraser-Liggett C. M. Knight R. Gordon J. I. (2007). The human microbiome project. Nature 449:804. 10.1038/nature06244 17943116 PMC3709439

[B32] Wilmoth J. L. Doak P. W. Timm A. Halsted M. Anderson J. D. Ginovart M.. (2018). A microfluidics and agent-based modeling framework for investigating spatial organization in bacterial colonies: the case of *Pseudomonas aeruginosa* and h1-type vi secretion interactions. Front. Microbiol. 9:33. 10.3389/fmicb.2018.00033 29467721 PMC5808251

[B33] Zapién-Campos R. Olmedo-Álvarez G. Santillán M. (2015). Antagonistic interactions are sufficient to explain self-assemblage of bacterial communities in a homogeneous environment: a computational modeling approach. Front. Microbiol. 6:489. 10.3389/fmicb.2015.00489 26052318 PMC4440403

